# The transcription factor BBX regulates phosphate homeostasis through the modulation of FGF23

**DOI:** 10.1038/s12276-024-01341-9

**Published:** 2024-11-01

**Authors:** Su Jeong Lee, Ju Ang Kim, Hye Jung Ihn, Je-Yong Choi, Tae-Yub Kwon, Hong-In Shin, Eui-Sic Cho, Yong Chul Bae, Rulang Jiang, Jung-Eun Kim, Eui Kyun Park

**Affiliations:** 1 Department of Oral Pathology and Regenerative Medicine, School of Dentistry, Institute for Hard Tissue and Bio‑tooth Regeneration (IHBR), Kyungpook National University, Daegu, Republic of Korea; 2 Cell and Matrix Research Institute, Kyungpook National University, Daegu, Republic of Korea; 3 Department of Biochemistry and Cell Biology, School of Medicine, Kyungpook National University, Daegu, Republic of Korea; 4 Department of Dental Biomaterials, School of Dentistry, Kyungpook National University, Daegu, Republic of Korea; 5 Cluster for Craniofacial Development and Regeneration Research, Institute of Oral Biosciences, School of Dentistry, Jeonbuk National University, Jeonju, Republic of Korea; 6 Department of Oral Anatomy and Neurobiology, School of Dentistry, Kyungpook National University, Daegu, Republic of Korea; 7 Division of Developmental Biology, Cincinnati Children’s Hospital Medical Center, Ohio, TX USA; 8 Department of Molecular Medicine, School of Medicine, Kyungpook National University, Daegu, Republic of Korea

**Keywords:** Bone development, Mechanisms of disease

## Abstract

Fibroblast growth factor 23 (FGF23) plays an important role in phosphate homeostasis, and increased FGF23 levels result in hypophosphatemia; however, the molecular mechanism underlying increased FGF23 expression has not been fully elucidated. In this study, we found that mice lacking the bobby sox homolog (*Bbx*^*−/−*^) presented increased FGF23 expression and low phosphate levels in the serum and skeletal abnormalities such as a low bone mineral density (BMD) and bone volume (BV), as well as short and weak bones associated with low bone formation. Osteocyte-specific deletion of *Bbx* using Dmp-1-Cre resulted in similar skeletal abnormalities, elevated serum FGF23 levels, and reduced serum phosphate levels. In *Bbx*^*−/−*^ mice, the expression of sodium phosphate cotransporter 2a (*Npt2a*) and *Npt2c* in the kidney and *Npt2b* in the small intestine, which are negatively regulated by FGF23, was downregulated, leading to phosphate excretion/wasting and malabsorption. An in vitro *Fgf23* promoter analysis revealed that 1,25-dihydroxyvitamin D_3_ (1,25(OH)_2_D_3_)-induced transactivation of the *Fgf23* promoter was significantly inhibited by BBX overexpression, whereas it was increased following *Bbx* knockdown. Interestingly, 1,25(OH)_2_D_3_ induced an interaction of the 1,25(OH)_2_D_3_ receptor (VDR) with BBX and downregulated BBX protein levels. Cycloheximide (CHX) only partially downregulated BBX protein levels, indicating that 1,25(OH)_2_D_3_ regulates BBX protein stability. Furthermore, the ubiquitination of BBX followed by proteasomal degradation was required for the increase in *Fgf23* expression induced by 1,25(OH)_2_D_3_. Collectively, our data demonstrate that BBX negatively regulates *Fgf23* expression, and consequently, the ubiquitin-dependent proteasomal degradation of BBX is required for FGF23 expression, thereby regulating phosphate homeostasis and bone development in mice.

## BBX degradation drives FGF23 expression and compromises bone health

Phosphate, a vital component for various bodily functions, is regulated by a complex system involving hormones like FGF23. Researchers investigated how the absence of a gene called Bobby sox homolog (BBX) impacts phosphate balance and bone health. Researchers created BBX-deficient mice, and measured various factors, including bone mineral density and strength, as well as the impact of BBX on phosphate-regulating hormones. They found that lack of BBX in mice leads to lower phosphate levels, weaker bones, and increased FGF23 levels, disrupting phosphate balance. Additionally, they discovered that an active form of vitamin D_3_ lowers BBX levels through a process called ubiquitin-dependent proteasomal degradation, which is important for controlling FGF23. The study concluded that BBX deficiency causes significant bone weakness and disrupts phosphate levels by increasing FGF23. This summary was initially drafted using artificial intelligence, then revised and fact-checked by the author.

## Introduction

Phosphate is a fundamental mineral component required for various biological processes, including energy metabolism, cellular events, bone development, and mineralization in the body^1,2^. Phosphate homeostasis is regulated by the complex systemic endocrine control of intestinal phosphate absorption, renal reabsorption, and long-term skeletal storage^3^.

Endocrine factors, including fibroblast growth factor 23 (FGF23), 1,25-dihydroxyvitamin D_3_ [1,25(OH)_2_D_3_], and parathyroid hormone (PTH), regulate serum phosphate levels through a negative feedback loop^3–5^. PTH reduces the serum phosphate concentration by downregulating sodium phosphate cotransporter (NPT) 2a and NPT2c on the apical surface of the renal proximal tubule^6^. PTH also upregulates 1α-hydroxylase (CYP27B1), thereby increasing the production of 1,25(OH)_2_D_3_ in the kidney^7^. Subsequently, 1,25(OH)_2_D_3_ stimulates phosphate absorption in the intestine, reduces PTH production in the parathyroid gland, and increases serum FGF23 levels^8^. FGF23, a member of the FGF19 subfamily of FGFs, is predominantly secreted by osteocytes and osteoblasts in the bone. FGF23 promotes the renal excretion of phosphate by reducing its reabsorption resulting from the suppression of *NPT2a* and *NPT2c* expression in the renal proximal tubule^9–11^. Simultaneously, FGF23 decreases 1,25(OH)_2_D_3_ levels by downregulating the expression of *CYP27B1*, which encodes a key enzyme involved in its biosynthesis, and upregulating the expression of *CYP24A1*, which is an enzyme responsible for its catabolism^12,13^. An increase in circulating active FGF23 levels causes hypophosphatemia with significantly low or standard levels of 1,25(OH)_2_D_3_ and low PTH levels^14–16^.

Hypophosphatemia is a condition in which low serum phosphate levels affect multiple organs, including bone^17,18^. The clinical manifestations of hypophosphatemia can vary depending on its severity, the time of onset (acute or chronic), and individual age^2^. Hypophosphatemia is commonly associated with rickets, which is a defective condition of bone mineralization that occurs in children as a result of dysregulated phosphate transport and/or excessive serum FGF23 levels^19^. Most cases of hypophosphatemic rickets are associated with increased serum FGF23 levels^19^. The major clinical manifestations of hypophosphatemic rickets in children include growth retardation, bone weakness and deformity, and short stature^18,20^. Although various types of hypophosphatemic rickets are associated with FGF23, the underlying molecular mechanism and various causative factors for excessive FGF23 production remain unclear.

Bobby sox homolog (BBX) is a transcription factor that contains a high mobility group (HMG) box domain^21^. HMG box family members include the Sry-related Sox and TCF/LEF genes, which play important roles in skeletal diseases, development, and Wnt signaling^21,22^. BBX is also known as HBP2 and was originally identified as a factor that promotes the G1/S transition in yeast^23^. It is evolutionarily conserved in various organisms from worms to mammals^21^. In *Bbx*-null rats, the number and motility of epididymal spermatozoa are reduced through apoptosis, suggesting that BBX regulates the survival of postmitotic spermatids and supports spermiogenesis and fertility in male rats^24^. Previously, we reported that BBX is upregulated during the odontoblast differentiation of human dental pulp stem cells and contributes to odontoblast differentiation in vitro^25^. We also documented that *Bbx* disruption decreases root elongation and mechanical strength in mice^26^. In addition, flexible and weak bone phenotypes have been reported in *Bbx*-deficient mice^27^. These results suggest that BBX may be involved in the regulation of tooth and bone development and mechanical strength; however, the role of BBX in hard tissue development and mechanical strength and the underlying molecular mechanism leading to abnormal bone development are unknown.

In the present study, we examined the phenotypic characteristics of skeletal regions in both conventional *Bbx*-deficient mice and osteoblast/osteocyte-specific *Bbx*-deficient mice and identified the molecular mechanism underlying the phenotypic and mechanical alterations induced by *Bbx* deficiency.

## Materials and methods

### Animals and skeletal preparation

All animal experiments were approved by the Animal Care and Use Committee for Research at Kyungpook National University and were conducted in accordance with the guidelines for the care and use of laboratory animals (KNU-2018-0048 and KNU 2021-0180). *Bbx*^*−/−*^ mice were generated by breeding the EUCOMM mouse strain C57BL/6N-Bbx^tm1a (EUCOMM)Wtsi/+^ (Wellcome Sanger Institute, Hinxton, Cambridgeshire, UK) with the Flp recombinase and protamine-Cre transgenic mouse strains, as described previously^26^. *Bbx*-deficient mice harbor a *Bbx* allele in which exon 6 is flanked by loxP sites. Genotyping was performed by PCR using tail genomic DNA. Bbx deletion was detected with forward 5′-CCACAAACAAGCCTGTGAAA-3′ and reverse CCAACCATTGGGTGTGTGTA-3′ primers. *Bbx*^*f/f*^*;Dmp1-Cre* mice were generated by breeding *Dentin matrix protein 1-Cre* (*Dmp1-Cre*) transgenic mice with our *Bbx*^*f/f*^ mice to delete Bbx in the osteoblast/osteocyte population via the Cre-Lox system. The following primers were used for PCR genotyping: *Bbx*^*f/x*^ forward, 5′-GGTTGCTGGTCCTAGCGTTC-3′; *Bbx*^*f/x*^ reverse, 5′-GCACATAGCACAGAGGCACAG-3′; *Dmp1-Cre* forward, 5′-ATCCGAAAAGAAAACGTTGA-3′; and *Dmp1-Cre* reverse, 5′-ATCCAGGTTACGGATATAGT-3′. The mice were fed a standard rodent chow diet, and their body weights were measured daily during the experimental period. The gross morphology of the skeleton of newborn mice (P0) was examined using alizarin red S/alcian blue (Sigma-Aldrich, Saint Louis, MO, USA) staining, as previously described^28^. Briefly, whole skeletons were fixed with 95% ethanol followed by staining with alcian blue and alizarin red S to visualize the cartilage and bone, respectively. The stained skeletons were observed under a stereomicroscope.

### Bone parameter analysis and mechanical tests

For analysis of bone morphology and characteristics, the mice were sacrificed, and their skulls, femurs, vertebrae, and ilium were dissected and fixed with 4% paraformaldehyde (PFA). The fixed bones were scanned using a SkyScan 1272 high-resolution microcomputed tomography (micro-CT) system (Bruker, Billerica, MA, USA; source voltage of 60 kV, current of 166 μA, and resolution of 8–10 μm). All parameters, including bone mineral density (BMD), bone volume (BV), bone volume/total volume (BV/TV), trabecular thickness (Tb.Th), trabecular number (Tb.N), trabecular space (Tb.Sp), cortical bone thickness (Ct.Th), cortical bone area (Ct.Ar), mean polar moment of inertia (MMI), eccentricity (Ecc), and cortical bone cross-sectional thickness (Ct.Cs.Th), were assessed using CTAn software (Bruker)^29^. Data viewer software was used to measure the femur length. Bone strength was measured using a 3-point bending test on rehydrated 4-week-old tibial shafts to analyze the mechanical properties of bone in *Bbx*^*+/*+^ and *Bbx*^*−/−*^ mice. The bones were horizontally positioned on two support points at 8-mm intervals, and a force (loading rate, 1 mm/min) was applied to the upper part at the mid-diaphysis until fracture. Load and displacement values were measured during the test and saved in a data file (Instron3343, Norwood, MA, USA).

### Histological analysis and immunohistochemistry

Bone and other tissues were fixed with 4% PFA in phosphate-buffered saline at 4 °C overnight. For bone histology, the fixed bone tissues were decalcified in 10% ethylenediaminetetraacetic acid for 2 weeks. Thereafter, the tissues were dehydrated with ethanol, embedded in paraffin, and serially sectioned at a 6-μm thickness. Hematoxylin and eosin (H&E) staining was conducted using a previously described procedure^30^. The osteoblast surface per bone surface (Obs/BS) was measured using the i-solution program (IMT i-Solution Inc., Riverton, UT, USA). For immunostaining, antigen retrieval was conducted at 90 °C for 20 min in 10 mM sodium citrate (pH 6.0). The sections were then incubated with an antibody against FGF23 (Bioss Antibodies Inc., Woburn, MA, USA; BS-5768R, 1:2000 dilution) at 4 °C overnight, followed by an incubation with biotinylated mouse anti-rabbit IgG (Santa Cruz Biotechnology, Inc., Dallas, TX, USA; SC-2491, 1:500 dilution). Detection was performed using a DAB Reagent Kit (Abcam, Cambridge, Cambridgeshire, UK; ab103723) according to the manufacturer’s instructions. Images were acquired using a light microscope (Leica Microsystems, Wetzlar, Hessen, Germany).

### Quantitative real-time PCR

Total RNA was extracted from the femur, kidney, or small intestine using TRIzol (BioScience Technology, Daegu, Korea), as described previously^29^. Complementary DNA was synthesized using Superior Script II reverse transcriptase (Invitrogen, Waltham, MA, USA). Then, qRT‒PCR was performed using SYBR Master Mix (Takara Bio, Inc., Kusatsu, Shiga, Japan) in a LightCycler 1.5 (Roche Diagnostics, Basel, Basel-Stadt, Switzerland) according to the manufacturer’s protocol. GAPDH was used as an internal reference for each sample and as the loading control. All primer sequences are listed in Supplementary Table 1.

### Western blot and immunoprecipitation

Protein samples were extracted using RIPA buffer supplemented with protease and phosphatase inhibitors (GenDEPOT, Altair, TX, USA). The immunoblot analysis was performed by resolving the proteins on SDS‒PAGE gels followed by transfer to nitrocellulose membranes (Cytiva, Marlborough, MA, USA). The membranes were incubated with a blocking solution (TBS-T containing 5% nonfat milk powder) for 1 h. Then, the membranes were incubated with primary antibodies, anti-BBX (Proteintech Group, Inc., Rosemont, IL, USA ; 17254-1-AP, 1:1000 dilution), anti-VDR (Abcam; ab109234, 1 μg for immunoprecipitation and 1:1000 dilution for western blot), anti-actin (Bioworld Technology, Inc., Bloomington, MN, USA; AP0060, 1:5000 dilution), anti-Flag (Sigma-Aldrich; F7425, 1 μg for immunoprecipitation and 1:1000 dilution for western blot), and anti-ubiquitin (Enzo Life Sciences, Inc., Farmingdale, NY, USA; BML-PW8810, 1:1000 dilution), followed by an incubation with secondary antibodies (Thermo Fisher Scientific, Inc., Waltham, MA, USA; #31460-rabbit, #31430-mouse). Chemiluminescence was generated using WesternBright ECL (Advansta Inc., San Jose, CA, USA) and detected using an Azure C600 system (Azure Biosystems, Inc., Dublin, CA, USA). For immunoprecipitation, protein lysates (600 μg) were precleared with control agarose beads (Thermo Fisher Scientific, Inc.; 26150) and incubated with anti-Flag or anti-VDR at 4 °C overnight with rotation and with Protein-A/G agarose (Thermo Fisher Scientific, Inc.; 20423) for 2 h. After several washes, the immunoprecipitates were heated at 100 °C for 5 min in SDS‒PAGE sample buffer, followed by SDS‒PAGE.

### Double fluorescence labeling

For dynamic histomorphometric measurements of bone formation, filter-sterilized calcein (Sigma-Aldrich; C0875, 30 mg/kg) dissolved in 2% sodium bicarbonate (pH 7.0) was injected intraperitoneally at 7 days and 3 days before harvest. The tibial shafts were dissected and dehydrated with an ascending ethanol series and embedded undecalcified in methyl methacrylate (MMA) using Technovit 9100 (Heraeus Kulzer, Hanau, Hesse, Germany). Each sample was cut into 7-μm sections to assess the mineral apposition rate (MAR) and bone formation rate/bone surface (BFR/BS), and sections were observed using a fluorescence microscope (Leica Microsystems).

### Acid etching and scanning electron microscopy (SEM)

For ultrastructural analysis, tibia samples were embedded in MMA without preliminary decalcification. The MMA-embedded samples were then acid-etched with 37% phosphoric acid for 10 s, washed twice with water, treated with 5% sodium hypochlorite for 3 min, and rinsed with water again. The samples were then coated with platinum and observed via SEM (Hitachi, Chiyoda, Tokyo, Japan; SU8220).

### Serum and urine biochemical analyses

The mice were anesthetized with 20 mL/kg of a 2% tribromoethanol (Avertin) solution, and blood was collected via cardiac puncture. The serum was prepared and stored at −80 °C until use. Urine was collected for 24 h in a metabolic cage. The phosphate concentrations in the serum and urine were measured using a phosphate assay kit (Abcam; ab65622), and the serum calcium concentration was measured using a Quantichrom Calcium Assay Kit (BioAssay Systems, Hayward, CA, USA; DICA-500). The actual FGF23, C-terminal FGF23, and PTH levels in the serum were quantified using a mouse FGF23 (intact) ELISA Kit (Immutopics, Inc., San Clemente, CA, USA; #60-6800), a FGF23 (C-Term) ELISA Kit (Immutopics, Inc.; #60-6300), and a mouse/rat intact PTH ELISA Kit (Mybiosource, San Diego, CA, USA; MBS704631). The 1,25(OH)_2_D_3_ levels were determined using an ELISA kit (Mybiosource; MBS2602146) according to the manufacturer’s instructions.

### DNA cloning and reporter assays

The *BBX* expression constructs were created by cloning full-length human and mouse *BBX* cDNAs into the pCDNA3 vector between the *Eco*RI and *Xho*I and *Kpn*I and *Xho*I sites, respectively. The Flag-tagged full-length *BBX* cDNA was subsequently cloned and inserted into the pHJ-1 vector between the *Xho*I and *Kpn*I sites. Human and mouse *VDR* cDNAs were cloned and inserted into the pCDNA3 vector between the *Eco*RI and *Cla*I and *BamH*I and *Xho*I sites, respectively. Several constructs containing different lengths of the mouse *Fgf23* promoter region were generated and cloned and inserted into the pGL2-basic vector (Promega) between the *Kpn*I and *Xho*I restriction sites: P2.0 FGF23 promoter (−2 K to −1), P1.3 FGF23 promoter (−1.3 K to −1), P1.0 FGF23 promoter (−1.0 K to −1) and P0.6 FGF23 promoter (−0.6 K to −1). The forward primer 5′-TGGTACCTAGCTGTGGCTATGATTCATCC-3′ (−2 K), 5′-GGGTACCATCCATGTGCTTTACACGATGG-3′ (−1.3 K), 5′-TGGTACCGCATGCACAGAGCAATTTCTGC-3′ (−1.0 K), or 5′-GCGGTACCTATGACCATATATCAAGACACTTGCC-3′ (−0.6 K) and reverse primer 5′-GCCTCGAGTGCACAGCACTGAGTGGCTAATGC-3′ (−1) were used to amplify the FGF23 promoters from mouse genomic DNA via PCR^8^. MC3T3-E1 cells were transfected with the indicated *Fgf23* promoter construct, *BBX* expression vector, or *Bbx* shRNA (Sigma-Aldrich) using FuGENE 6 (Roche Diagnostics) to examine the activity of the FGF23 promoter. The firefly/Renilla luciferase activities were analyzed using a dual-luciferase reporter assay system (Promega, Madison, WI, USA).

### Statistical analysis

All the results were analyzed using GraphPad Prism (GraphPad Software, Inc., San Diego, CA, USA) and are presented as the means ± standard deviations. Statistical analyses were performed using two-tailed Student’s *t-*test and one-way or two-way ANOVA. Values were considered statistically significant at *p* values < 0.05, *p* < 0.01 or *p* < 0.001.

## Results

### Postnatal growth retardation and skeletal alterations in *Bbx-*deficient mice

**Fig. 1:**
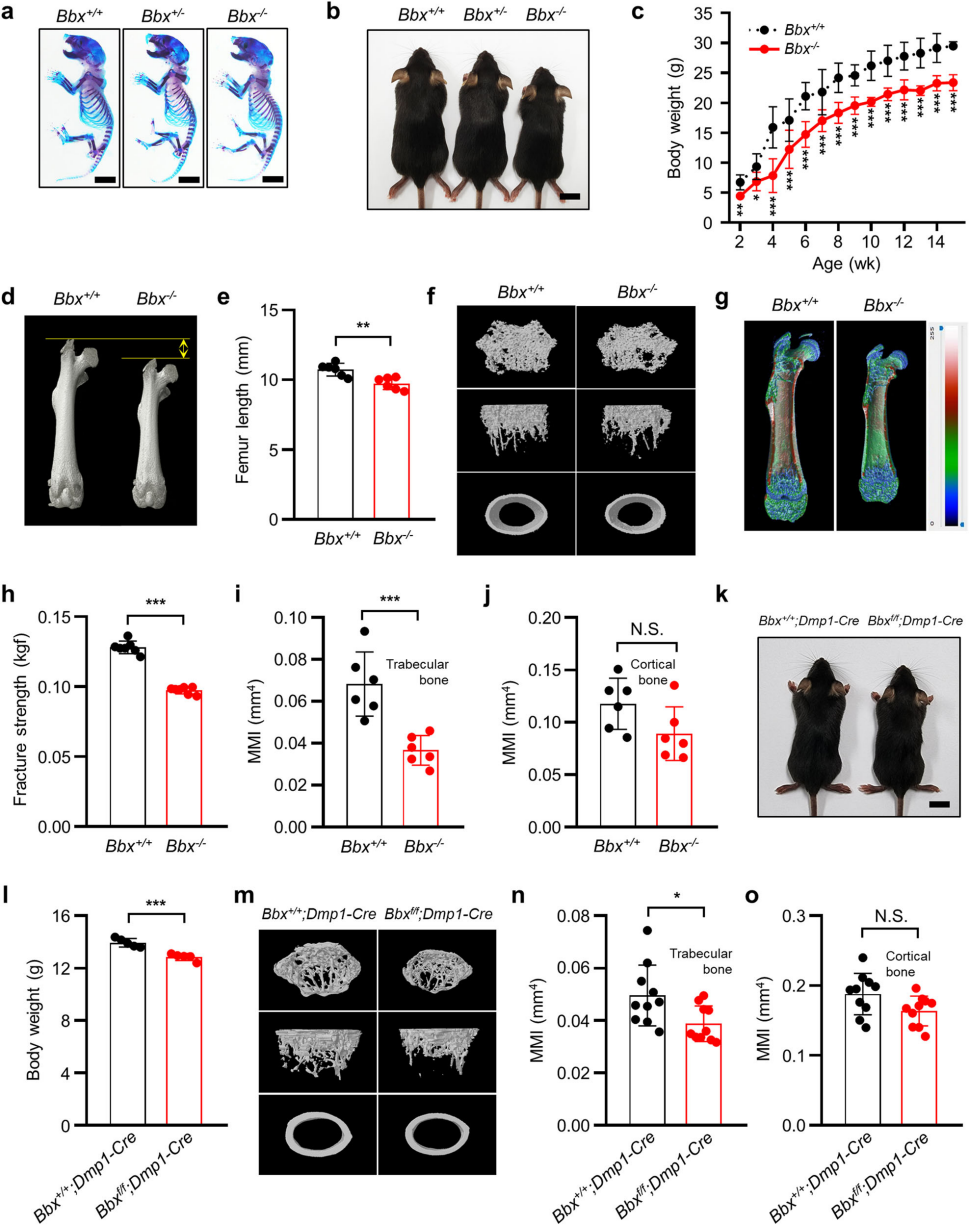
BBX disruption delays postnatal growth and alters the morphological and physical features of mice. **a** Morphology of the skeleton and cartilage of *Bbx*^*+/+*^, *Bbx*^*+/−*^, and *Bbx*^*−/−*^ mice at P0. P0 embryos were stained with alizarin red/alcian blue. Scale bars, 5 mm **b** Gross appearance of 4-week-old *Bbx*^*+/+*^, *Bbx*^*+/−*^, and *Bbx*^*−/−*^ mice. Scale bars, 1 cm. **c** Growth curves of *Bbx*^*+/+*^ and *Bbx*^*−/−*^ mice. At each time point, the body weights of the *Bbx*^*+/+*^ and *Bbx*^*−/−*^ mice were measured (*n* = 10). **d** Representative micro-CT images of femurs from 4-week-old *Bbx*^*+/+*^ and *Bbx*^*−/−*^ mice. **e** Comparison of the mean femur length in *Bbx*^*+/+*^ and *Bbx*^*−/−*^ mice using CTVox software (*n* = 6). **f** Representative micro-CT images of the femurs of 4-week-old *Bbx*^*+/+*^ and *Bbx*^*−/−*^ mice. **g** Pseudocolor images of the femurs of 4-week-old *Bbx*^*+/+*^ and *Bbx*^*−/−*^ mice. The color corresponds to the degree of BMC. A lower BMC is shown in blue and green, whereas a higher BMC is shown in red. **h** A three-point bending test was performed to determine the fracture strength of the tibias of *Bbx*^*+/+*^ and *Bbx*^*−/−*^ mice (*n* = 7). **i**, **j** The MMI was measured in the trabecular (**i**) and cortical (**j**) bones of the femurs of *Bbx*^*+/+*^ and *Bbx*^*−/−*^ mice using CTAn (*n* = 6). **k**, **l** Gross appearances and body weights of 4-week-old *Bbx*^*+/+*^*;Dmp1-Cre* and *Bbx*^*f/f*^*;Dmp1-Cre* mice (*n* = 5). **m** Representative micro-CT images of femurs from 4-week-old *Bbx*^*+/+*^*;Dmp1-Cre* and *Bbx*^*f/f*^*;Dmp1-Cre* mice. **n**, **o** The MMI was measured in the trabecular (**n**) and cortical (**m**) bones of femurs from *Bbx*^*+/+*^*;Dmp1-Cre* and *Bbx*^*f/f*^*;Dmp1-Cre* mice using CTAn (*n* = 10). The data are presented as the means ± SDs. Each dot represents one independent experiment. ^*^p < 0.05, ^**^p < 0.01, and ^***^p < 0.001 as determined by an unpaired Student’s *t-*test between the two indicated genotypes. N.S. indicates no significant difference compared with control mice. Male mice were used in all the experiments.

*Bbx*-deficient mice were generated as described previously^26^, and *Bbx*^*f/f*^*;Dmp1-Cre mice* were generated to determine the function of BBX in bone development in vivo. Exon 6 of *Bbx* was deleted in the knockout mice, and *Bbx* disruption was confirmed by reverse transcription‒polymerase chain reaction (RT–PCR) using a primer set that amplifies exon 6 (Supplementary Fig. 1a, b). At birth (P0), the *Bbx*^*−/−*^ mice appeared healthy and phenotypically indistinguishable from their wild-type littermates (*Bbx*^*+/+*^), as assessed by alizarin red S/alcian blue staining of the embryos (Fig. 1a); however, the postnatal body size and body weight of the *Bbx*^*−/−*^ mice were progressively lower than those of the *Bbx*^*+/+*^ mice (Fig. 1b, c). Therefore, we examined the postnatal characteristics of bone in *Bbx*-deficient and wild-type mice. Micro-CT analyses of 4-week-old mice revealed that the femur length of *Bbx*^*−/−*^ mice was significantly shorter (by 9.24% ± 1.80%) than that of *Bbx*^*+/+*^ mice (Fig. 1d, e). Moreover, micro-CT analyses of the bone microstructure and mass indicated that the structure of the trabecular bone was coarser in *Bbx*^*−/−*^ mice than in *Bbx*^*+/+*^ mice (Fig. 1f). The BMD, BV, and BV/TV were significantly decreased by 16.62% ± 3.71%, 33.76% ± 7.25%, and 25.99% ± 5.35%, respectively, in *Bbx*^*−/−*^ mice. Tb.Th and Tb.N were also significantly reduced in *Bbx*^*−/−*^ mice by 5.45% ± 1.40% and 21.72% ± 4.63%, respectively (Table 1). Cortical bone parameters were not significantly different between *Bbx*^*+/+*^ and *Bbx*^*−/−*^ mice (Table 1), although the femurs of *Bbx*^*−/−*^ mice were smaller and shorter than those of *Bbx*^*+/+*^ mice (Fig. 1d–f). Less pronounced changes were also observed in other skeletal areas, including the frontal bone, spine, and ilium (Supplementary Table 2). The mineralization patterns and mechanical properties of the femurs and tibias were examined. An analysis of three-dimensional pseudocolor images revealed that the diaphysis of the femur in *Bbx*^*−/−*^ mice was less mineralized than that in *Bbx*^*+/+*^ mice (Fig. 1g). A three-point bending test of the tibial shafts revealed that the fracture strength of bone in *Bbx*^*−/−*^ mice was significantly lower than that in *Bbx*^*+/+*^ mice (Fig. 1h). In addition, the MMI, which represents resistance to torsional deformation, of the trabecular bone was lower in *Bbx*^*−/−*^ mice than in *Bbx*^*+/+*^ mice (Fig. 1i). The MMI of the cortical bone was also decreased in *Bbx*^*−/−*^ mice, although the difference was not statistically significant (Fig. 1j). Growth and bone abnormalities were analyzed in *Bbx*^*+/+*^*;Dmp1-Cre* and *Bbx*^*f/f*^*;Dmp1-Cre* mice (Supplementary Fig. 1c, d). Compared with *Bbx*^*+/+*^*;Dmp1-Cre* control mice, four-week-old *Bbx*^*f/f*^*;Dmp1-Cre* mice exhibited significant reductions in body size and body weight (Fig. k, l). As expected, the three-dimensional images from micro-CT analyses revealed differences in the trabecular bone architecture of *Bbx*^*f/f*^*;Dmp1-Cre* mice (Fig. 1m). Compared with those in *Bbx*^*+/+*^*;Dmp1-Cre* mice, the bone BMD, BV, BV/TV, and Tb.N were all decreased by 16.47% ± 7.93%, 23.30% ± 15.03%, 18.27% ± 5.35%, and 14.42% ± 10.73%, respectively, in *Bbx*^*f/f*^*;Dmp1-Cre* mice (Table 2). The MMI of the trabecular bone was also decreased in *Bbx*^*f/f*^*;Dmp1-Cre* mice (Fig. 1n, o). The phenotypic alterations observed in the long bones were also evident in 8-week-old *Bbx*^*−/−*^ and *Bbx*^*f/f*^*;Dmp1-Cre* mice. In *Bbx*^*−/−*^ mice, femoral length and specific cortical bone parameters were significantly reduced, whereas trabecular bone parameters showed reductions that were not statistically significant (Supplementary Table 3 and Supplementary Fig. 2a). The MMI of the trabecular and cortical bone was also markedly reduced in *Bbx*^*−/−*^ mice compared with *Bbx*^*+/+*^ mice (Supplementary Table 3). In *Bbx*^*f/f*^*;Dmp1-Cre* mice, significant decreases in femoral length, trabecular bone parameters, and cortical thickness were observed. Other bone parameters showed a statistically insignificant reduction compared with *Bbx*^*+/+*^*;Dmp1-Cre* mice (Supplementary Table 3 and Supplementary Fig. 2b). These results indicate that *Bbx* deficiency causes significant postnatal growth retardation, characterized by decreased bone growth, bone parameters, and mechanical strength.

**Table 1:** Bone parameters of the femurs of 4-week-old wild-type and *Bbx*-deficient mice (±SD).

Bone	Genotype	BMD (g/mm^3^)	BV (mm^3^)	BV/TV (%)	Tb.Th (mm)	Tb.N (1/mm)	Tb.Sp (mm)
Trabecular bone	*Bbx*^*+/+*^	0.115 (0.011)	0.326 (0.075)	11.752 (1.699)	0.047 (0.003)	2.484 (0.274)	0.381 (0.051)
	*Bbx*^*−/−*^	0.095 (0.013)^*^	0.216 (0.058)^*^	8.697 (1.692)^**^	0.045 (0.001)^*^	1.944 (0.353)^*^	0.446 (0.065)

Bone	Genotype	BMD (g/mm^3^)	BV/TV (%)	Ct.Th (mm)	Ct.Ar (mm^2^)	Ecc	Ct.Cs.Th (mm)
Cortical bone	*Bbx*^*+/+*^	0.556 (0.009)	65.074 (0.990)	0.103 (0.004)	0.348 (0.033)	0.573 (0.022)	0.086 (0.004)
	*Bbx*^*−/−*^	0.540 (0.016)	63.408 (1.863)	0.096 (0.008)	0.303 (0.043)	0.555 (0.049)	0.080 (0.006)

An unpaired Student’s *t-*test was performed. ^*^p < 0.05 and ^**^p < 0.01.

**Table 2:** Bone parameters of the femurs of 4-week-old *Bbx*^*+/+*^*;Dmp1-Cre* and *Bbx*^*f/f*^*;Dmp1-Cre* mice (±SD).

Bone	Genotype	BMD (g/mm^3^)	BV (mm^3^)	BV/TV (%)	Tb.Th (mm)	Tb.N (1/mm)	Tb.Sp (mm)
Trabecular bone	*Bbx*^*+/+*^*;Dmp1-Cre*	0.108 (0.010)	0.440 (0.064)	17.162 (1.223)	0.087 (0.005)	1.985 (0.147)	0.380 (0.030)
	*Bbx*^*f/f*^*;Dmp1-Cre*	0.090 (0.009)^***^	0.337 (0.066)^**^	14.027 (2.705)^**^	0.082 (0.009)	1.699 (0.213)^**^	0.411 (0.032)^*^

Bone	Genotype	BMD (g/mm^3^)	BV/TV (%)	Ct.Th (mm)	Ct.Ar (mm^2^)	Ecc	Ct.Cs.Th (mm)
Cortical bone	*Bbx*^*+/+*^*;Dmp1-Cre*	0.653 (0.044)	40.265 (2.339)	0.159 (0.008)	0.542 (0.041)	0.568 (0.025)	0.139 (0.007)
	*Bbx*^*f/f*^*;Dmp1-Cre*	0.579 (0.034)^***^	39.683 (3.978)	0.153 (0.014)	0.499 (0.039)^*^	0.575 (0.027)	0.133 (0.012)

An unpaired Student’s *t-*test was performed. ^*^p < 0.05, ^**^p < 0.01, and ^***^p < 0.001.

### Reduced formation of bone and growth plates in *Bbx-*deficient mice

**Fig. 2:**
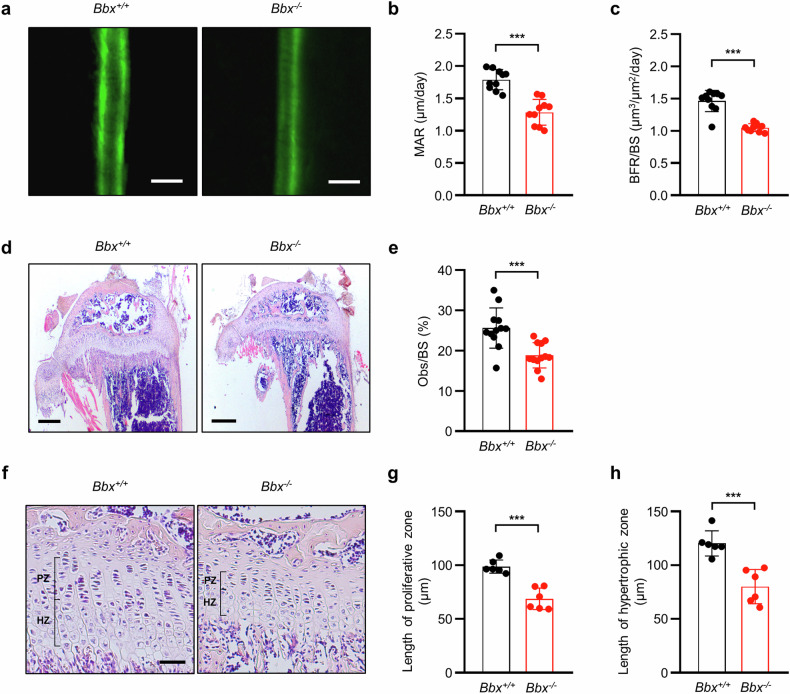
BBX disruption decreases bone formation, the number of osteoblasts, and growth plate length. **a** Representative fluorescence images of tibial cross-sections (mid-diaphysis area) from 4-week-old *Bbx*^*+/+*^ and *Bbx*^*−/−*^ mice. Bone formation was visualized using double calcein labeling. Scale bars, 10 μm. **b**, **c** MAR (**b**) and BFR/BS (**c**) at the mid-diaphysis of the tibial shafts from 4-week-old *Bbx*^*+/+*^ and *Bbx*^*−/−*^ mice were measured (*n* = 10). **d** H&E staining of sagittal tibial sections from 4-week-old *Bbx*^*+/+*^ and *Bbx*^*−/−*^ mice. Scale bars, 1 mm. **e** Quantitative histomorphometric analyses of the proximal tibia. Obs/BS was measured using i-solution (*n* = 12). **f** Higher magnification images of the growth plate area in (**d**). Scale bars, 50 μm. **g**, **h** The lengths of the proliferative chondrocyte zones (PZ) (**g**) and hypertrophic chondrocyte zones (HZ) (**h**) were measured in the growth plates of 4-week-old *Bbx*^*+/+*^ and *Bbx*^*−/−*^ mice. The data are presented as the means ± SDs. Each dot represents one independent experiment. ^***^p < 0.001, as determined by an unpaired Student’s *t-*test between the two indicated genotypes.

BMD, BV, and bone mechanical strength are closely associated with bone formation and resorption^31–33^. Because these bone parameters were significantly reduced in *Bbx*^*−/−*^ mice, we measured the BFR and MAR in nondecalcified histological sections of the tibia following calcein injections. As shown in Fig. 2a, the fluorescence image analysis revealed a smaller distance between labeled fronts in the tibial sections of *Bbx*^*−/−*^ mice. Similarly, the MAR and BFR in *Bbx*^*−/−*^ mice were decreased by 28.24% ± 3.90% and 23.07% ± 3.05%, respectively, compared with those in *Bbx*^*+/+*^ mice (Fig. 2b, c). Histological staining of the tibial shafts with H&E revealed that the areas of the proximal epiphysis and metaphysis and the osteoblast surface per bone surface were lower in *Bbx*^*−/−*^ mice than in *Bbx*^*+/+*^ mice (Fig. 2d, e), indicating that bone formation was significantly reduced in *Bbx*^*−/−*^ mice. Notably, osteoclast-specific tartrate-resistant acid phosphatase staining of tibial sections also revealed a significant reduction in the number of osteoclasts in *Bbx*^*−/−*^ mice (Supplementary Fig. 3a, b). Considering that the long bones of *Bbx*^*−/−*^ mice were significantly shorter than those of *Bbx*^*+/+*^ mice (Fig. 1d, e), the length of the growth plates was assessed. H&E staining of the tibia indicated that the proliferative and hypertrophic zones of the growth plate cartilage were thinner in the *Bbx*^*−/−*^ mice (Fig. 2f–h). These results suggest that bone formation by osteoblasts, growth plate formation by chondrocytes, and bone resorption by osteoclasts are significantly reduced due to *Bbx* deficiency. The reduced activities or numbers of associated cells may be attributed to the small, short, and mechanically weak bones in *Bbx*-deficient mice.

### Hypophosphatemia and increased serum FGF23 levels in *Bbx*-deficient mice

**Fig. 3:**
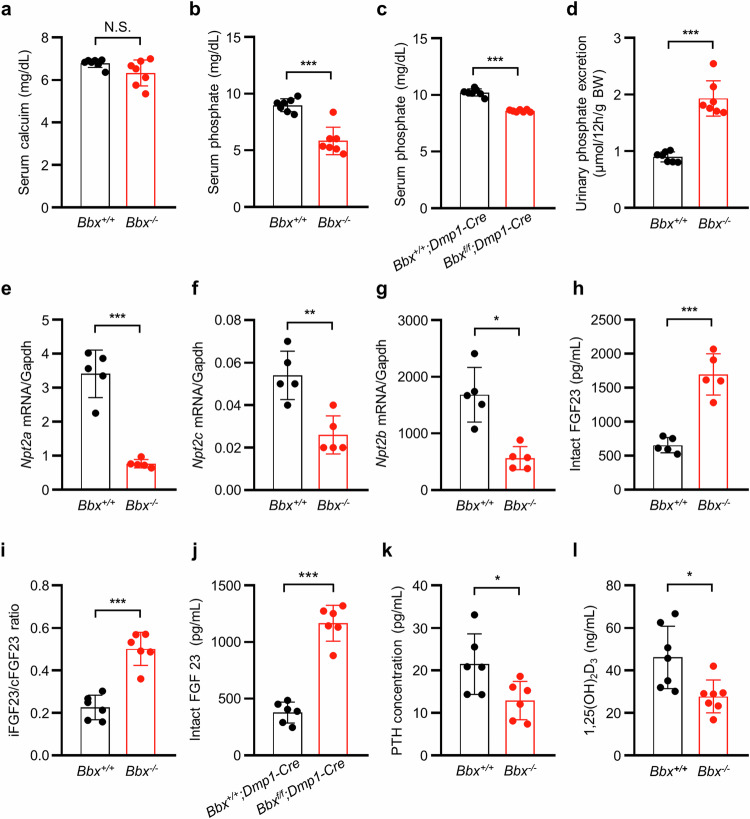
BBX disruption induces hypophosphatemia resulting from phosphate excretion and malabsorption via the upregulation of FGF23. Analyses of the serum levels of ions and regulatory factors, and tissue expression of ion transporters were performed in 4-week-old mice. **a**–**d** Serum levels of calcium (**a**) and phosphate (**b**) and urine concentrations of phosphate (**d**) were measured in samples collected from *Bbx*^*+/+*^ and *Bbx*^*−/−*^ mice (*n* = 7). Serum levels of phosphate (**c**) were measured in samples collected from *Bbx*^*+/+*^*;Dmp1-Cre* and *Bbx*^*f/f*^*;Dmp1-Cre* mice (*n* = 7). **e**‒**g** Renal *Npt2a* (**e**), *Npt2c* (**f**), and small intestinal *Npt2b* (**g**) mRNA levels were measured via qRT‒PCR (*n* = 5). **h**–**l** The intact FGF23 levels (**h**), iFGF23/cFGF23 ratio (**i**), PTH levels (**k**), and 1,25(OH)_2_D_3_ levels (**l**) were measured in serum samples from *Bbx*^*+/+*^ and *Bbx*^*−/−*^ mice (*n* = 5‒7), and the intact FGF23 levels (**j**) were measured in serum samples from *Bbx*^*+/+*^*;Dmp1-Cre* and *Bbx*^*f/f*^*;Dmp1-Cre* mice (*n* = 6). The data are presented as the means ± SDs. Each dot represents one independent experiment. ^*^p < 0.05, ^**^p < 0.01, and ^***^p < 0.001, as determined by an unpaired Student’s *t-*test between the two indicated genotypes. N.S. indicates no significant difference compared with control mice.

The mechanical strength of bone is related primarily to BV and bone quality^34–36^, and bone quality is dependent on the macro- and microarchitecture, minerals, and collagens^37,38^. Because *Bbx*-deficient mice have a low BV and BMD and weak mechanical strength, we determined whether the mineralization process was impaired in *Bbx-*deficient mice. We examined the serum levels of calcium, phosphate, and associated factors. Serum biochemistry revealed that calcium levels were not significantly different between *Bbx*^*+/+*^ and *Bbx*^*−/−*^ mice (Fig. 3a); however, serum phosphate levels were significantly lower (by 33.48% ± 5.92%) in *Bbx*^*−/−*^ mice than in *Bbx*^*+/+*^ mice (Fig. 3b). In *Bbx*^*f/f*^*;Dmp1-Cre* mice, the serum phosphate level was also 14.84% ± 1.10% lower than that in *Bbx*^*+/+*^*;Dmp1-Cre* mice (Fig. 3c). Low serum phosphate levels may be caused by excretion into the urine or malabsorption in the intestine. A urine analysis revealed that phosphate levels increased by 114.35% ± 17.14% in *Bbx*^*−/−*^ mice (Fig. 3d); however, histological sections of the kidney indicated no obvious structural abnormalities in *Bbx*^*−/−*^ mice (Supplementary Fig. 4a). Furthermore, we examined the expression of phosphate transporters involved in phosphate reabsorption in the kidney. *Npt2a* and *Npt2c* mRNA levels were significantly lower in the kidneys of *Bbx*^*−/−*^ mice than in those of *Bbx*^*+/+*^ mice (Fig. 3e, f). In addition, *Npt2b* expression was lower in the small intestine of *Bbx*^*−/−*^ mice than in that of *Bbx*^*+/+*^ mice (Fig. 3g). These results suggest that *Bbx* deficiency causes hypophosphatemia through phosphate excretion, wasting, and malabsorption because of the downregulation of phosphate transporters. Having established that *Bbx* deficiency causes hypophosphatemia, we examined putative BBX-mediated regulatory mechanisms for phosphate homeostasis. Because FGF23, PTH, and 1,25(OH)_2_D_3_ contribute to phosphate metabolism^39,40^, we measured the serum levels of these factors using enzyme-linked immunosorbent assays (ELISAs). As shown in Fig. 3h, i, the levels of intact FGF23 (the active form) and the ratio of intact FGF23/C-terminal FGF23 fragment (inactive form) were significantly increased (159.15% ± 28.51% and 122.16% ± 20.22%, respectively) in the serum of *Bbx*^*−/−*^ mice. Additionally, the level of intact FGF23 was markedly increased by 208.46% ± 42.00% in *Bbx*^*f/f*^*;Dmp1-Cre* mice compared with *Bbx*^*+/+*^*;Dmp1-Cre* mice (Fig. 3j). Moreover, the serum PTH and 1,25(OH)_2_D_3_ levels were significantly decreased by 39.86% ± 9.72% and 39.87% ± 8.55%, respectively, in *Bbx*^*−/−*^ mice (Fig. 3k, l). These results strongly suggest that *Bbx* deficiency results in increased serum FGF23 levels, which lead to hypophosphatemia caused by phosphate excretion, wasting and malabsorption in mice.

### Increased production of FGF23 in osteoblasts/osteocytes and decreased number and activity of osteocytes in *Bbx-*deficient mice

**Fig. 4:**
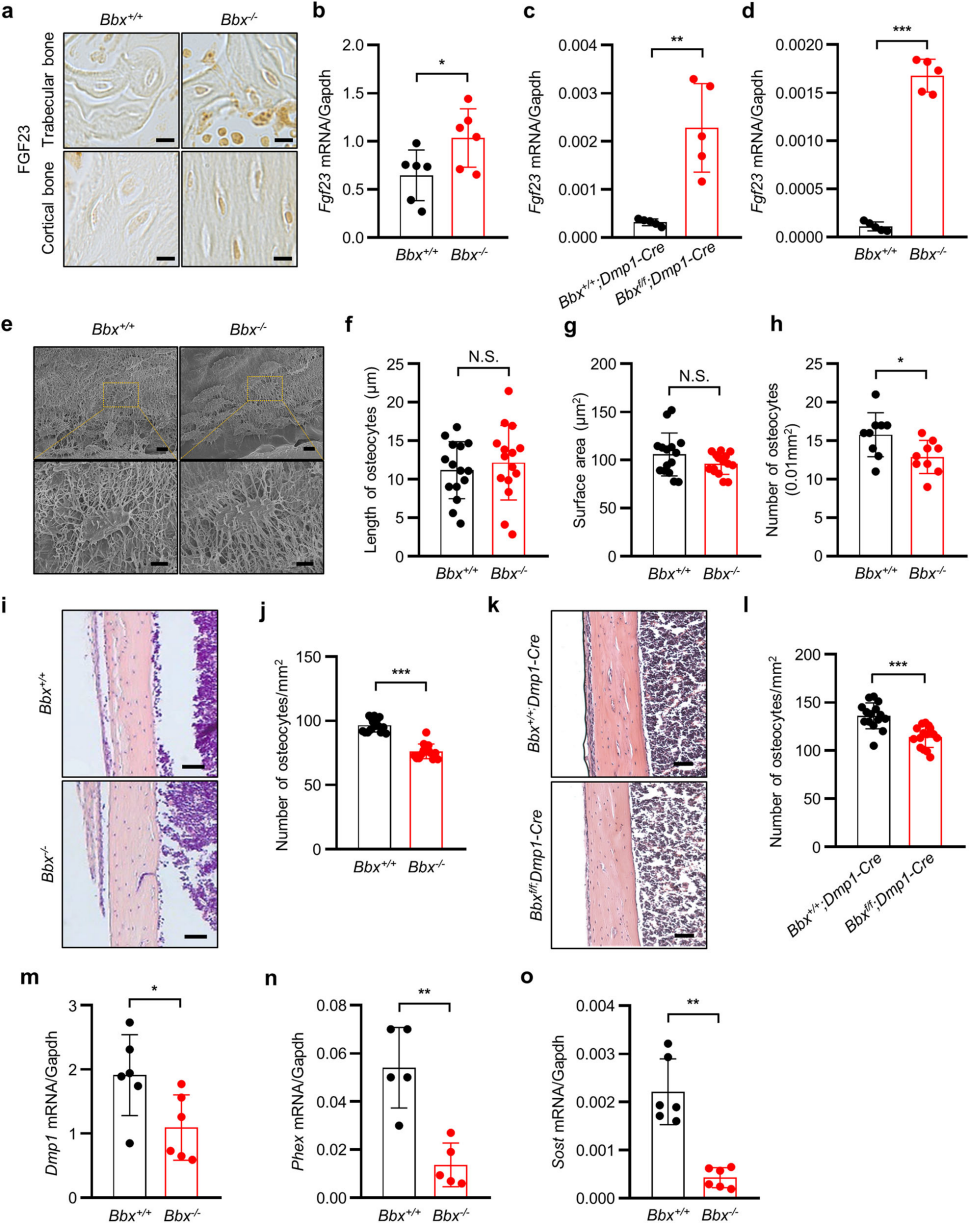
BBX disruption increases FGF23 production in osteoblasts and osteocytes and decreases the number and activity of osteocytes. **a** Immunohistochemical staining for FGF23 in osteocytes from the femurs of *Bbx*^*+/+*^ and *Bbx*^*−/−*^ mice. Scale bars, 10 μm (lower panel). **b** *Fgf23* mRNA expression in the femurs of *Bbx*^*+/+*^ and *Bbx*^*−/−*^ mice was measured via qRT‒PCR (*n* = 6). **c** The expression of the *Fgf23* mRNA in the femurs of *Bbx*^*+/+*^*;Dmp1-Cre* and *Bbx*^*f/f*^*;Dmp1-Cre* mice was measured (*n* = 5). **d** *Fgf23* mRNA expression was measured in BMSC-derived osteoblasts from *Bbx*^*+/+*^ and *Bbx*^*−/−*^ mice (*n* = 5). **e** SEM images of acid-etched cortical bone. Scale bars, 10 μm (upper panel) and 5 μm (lower panel). **f**–**h** Morphometric analyses of osteocytes. The length (**f**), surface area (**g**), and number (**h**) of osteocytes in the cortical bone of *Bbx*^*+/+*^ and *Bbx*^*−/−*^ mice were analyzed in SEM images (*n* = 9–15). **i** H&E staining of sagittal tibial sections from *Bbx*^*+/+*^ and *Bbx*^*−/−*^ mice. Scale bars, 50 μm. **j** The number of osteocytes per bone surface (mm^2^) from *Bbx*^*+/+*^ and *Bbx*^*−/−*^ mice in (**i**) was determined (*n* = 15). **k** H&E staining of sagittal tibial sections from *Bbx*^*+/+*^*;Dmp1-Cre* and *Bbx*^*f/f*^*;Dmp1-Cre* mice. Scale bars, 50 μm. **l** The number of osteocytes per bone surface (mm^2^) from *Bbx*^*+/+*^*;Dmp1-Cre* and *Bbx*^*f/f*^*;Dmp1-Cre* mice in (**k**) was analyzed (*n* = 15). **m**–**o** *Dmp1* (**m**), *Phex* (**n**), and *Sost* (**o**) mRNA expression levels were measured in the femurs of *Bbx*^*+/+*^ and *Bbx*^*−/−*^ mice (*n* = 5‒6). The data are presented as the means ± SDs. Each dot represents one independent experiment. ^*^p < 0.05, ^**^p < 0.01, and ^***^p < 0.001, as determined by an unpaired Student’s *t-*test between the two indicated genotypes. N.S. indicates no significant difference compared with control mice.

The role of FGF23 in phosphate homeostasis is well known, and its marked elevation has been detected in the osteocytes of patients with hypophosphatemia^41,42^. Therefore, we examined FGF23 expression in osteoblasts/osteocytes from *Bbx*-deficient mice. Immunohistochemical staining of the femur revealed higher FGF23 expression in the osteocytes of *Bbx*^*−/−*^ mice than in those of *Bbx*^*+/+*^ mice (Fig. 4a). Furthermore, *Fgf23* mRNA expression in the femur was also increased in both *Bbx*^*−/−*^ and *Bbx*^*f/f*^*;Dmp1-Cre* mice (Fig. 4b, c). Cell culture experiments revealed that *Fgf23* mRNA expression was markedly increased during the osteoblast differentiation of bone marrow mesenchymal stem cells (BMSCs) derived from *Bbx*^*−/−*^ mice compared with cells from *Bbx*^*+/+*^ mice (Fig. 4d). These results suggest that osteoblasts and osteocytes in *Bbx*^*−/−*^ mice generate more FGF23 than those in *Bbx*^*+/+*^ mice do. Furthermore, we determined whether alterations in the structure or number of osteocytes are associated with increased FGF23 production. Acid etching followed by SEM revealed that the length and surface area of osteocytes in *Bbx*^*−/−*^ mice were not significantly different from those in *Bbx*^*+/+*^ mice (Fig. 4e–g); however, the number of osteocytes in the cortical bone was significantly lower (by 19.92%) in *Bbx*^*−/−*^ mice than in *Bbx*^*+/+*^ mice (Fig. 4h). A histological examination of cortical bone sections also revealed significantly fewer osteocytes in *Bbx*^*−/−*^ mice than in *Bbx*^*+/+*^ mice (Fig. 4i, j). In *Bbx*^*f/f*^*;Dmp1-Cre* mice, a noticeable decrease in the number of osteocytes in the histological sections of cortical bone was observed (Fig. 4k, l). In addition, the expression of osteocyte marker genes, including dentin matrix protein 1 (*Dmp1*), phosphate regulating endopeptidase homolog, X-linked (*Phex*), and sclerostin (*Sost*), was markedly decreased (Fig. 4m–o), whereas no significant differences were detected in the expression of the osteoblast marker genes in *Bbx*^*−/−*^ mice compared with *Bbx*^*+/+*^ mice (Supplementary Fig. 5a–f). These results suggest that *Bbx* deficiency decreases the number of osteocytes and attenuates their expression of related genes.

### Transcriptional regulation of the FGF23 promoter by BBX

**Fig. 5:**
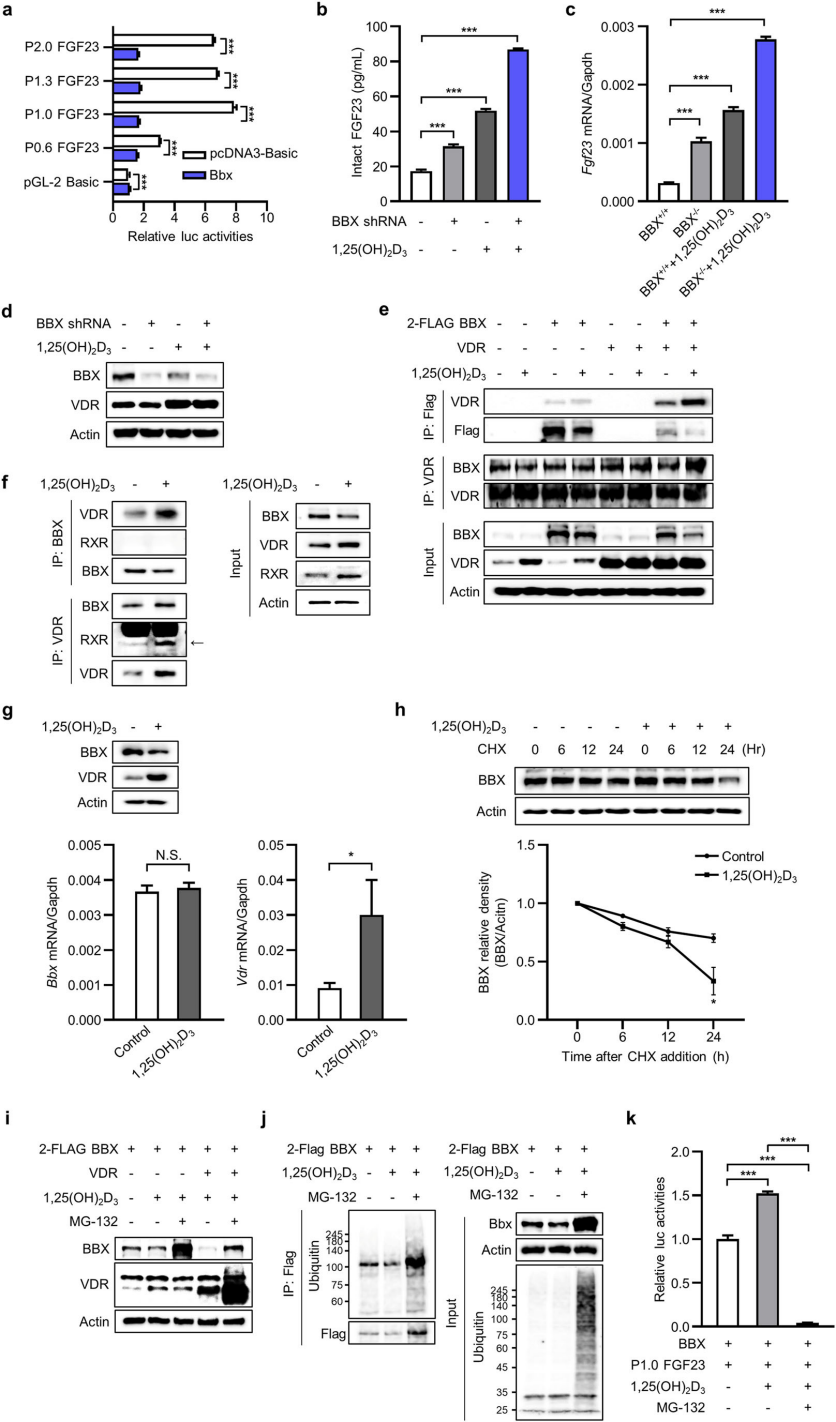
BBX negatively regulates the 1,25(OH)_2_D_3_-mediated transcriptional activity of FGF23. **a** MC3T3-E1 cells were cotransfected with the *BBX* cDNA in the pcDNA3-Basic vector (150 ng) and *Fgf23* promoter-firefly luciferase construct in the pGL-2 Basic plasmid (150 ng), incubated with 1,25(OH)_2_D_3_ (20 nM) for 24 h, and dual-luciferase assays were performed. **b** MC3T3-E1 cells were transfected with the *Bbx* shRNA or scrambled shRNA and incubated with or without 1,25(OH)_2_D_3_ (20 nM) for 24 h, after which FGF23 production was measured. The intact FGF23 level in the culture medium was quantified using an ELISA to detect intact FGF23. **c** *Fgf23* mRNA expression was measured in response to 1,25(OH)_2_D_3_ treatment in BMSC-derived osteoblasts from *Bbx*^*+/+*^ and *Bbx*^*−/−*^ mice. **d** MC3T3-E1 cells were transfected with the scrambled or *Bbx* shRNA and treated with or without 1,25(OH)_2_D_3_ (20 nM) for 24 h. BBX levels and 1,25(OH)_2_D_3_ receptor (VDR) protein levels were assessed via immunoblot analysis. **e** Interaction between BBX and VDR. MC3T3-E1 cells were transfected with 2-Flag-tagged *BBX* and *VDR* plasmids and immunoprecipitated with anti-Flag or anti-VDR antibodies. The interacting proteins were detected with the corresponding antibodies. **f** Interactions between endogenous BBX, VDR, and RXR. MC3T3-E1 cells were treated with 1,25(OH)_2_D_3_ (20 nM) for 30 min. Endogenous BBX and VDR were immunoprecipitated with anti-BBX or anti-VDR antibodies, and interacting protein levels were analyzed by immunoblotting. **g** Effects of 1,25(OH)_2_D_3_ on BBX protein downregulation and BBX mRNA levels. MC3T3-E1 cells were treated with 1,25(OH)_2_D_3_ (20 nM) for 24 h and analyzed by immunoblotting and qRT‒PCR. **h** Effect of 1,25(OH)_2_D_3_ treatment on BBX protein expression in the presence of CHX. MC3T3-E1 cells were treated with CHX (10 μM) for 1 h, followed by treatment with 1,25(OH)_2_D_3_ (20 nM) for each time course. **i** Analysis of the proteasomal degradation of BBX. MC3T3-E1 cells were transfected with the indicated plasmids and treated with or without MG-132 (10 μM) for 1 h, followed by treatment with 1,25(OH)_2_D_3_ (20 nM) for 12 h. The cell lysates were subjected to immunoblotting with the indicated antibodies. **j** 1,25(OH)_2_D_3_ increases BBX ubiquitination. MC3T3-E1 cells transfected with 2-Flag-*BBX* cDNA were treated with MG-132 (10 μM) for 1 h, followed by treatment with 1,25(OH)_2_D_3_ (20 nM) for 12 h. The Flag tag was immunoprecipitated from whole-cell lysates and detected with a ubiquitin antibody by immunoblotting. **k** MC3T3-E1 cells were cotransfected with the *BBX* cDNA and P1.0 FGF23 promoter-firefly luciferase plasmid and treated with MG-132 (10 μM) for 1 h, followed by treatment with 1,25(OH)_2_D_3_ (20 nM) for 12 h. The relative luciferase activity was measured. Actin was used as a loading control. The data are presented as the means ± SDs. ^***^p < 0.001, as determined by one-way or two-way ANOVA.

We measured FGF23 expression using the following *Fgf23* promoter constructs to identify the mechanism underlying the regulation of *Fgf23* expression by BBX: P2.0 FGF23 (ranging from −2 K to −1), P1.3 FGF23 (ranging from −1.3 K to −1), P1.0 FGF23 (ranging from −1 K to −1), and P0.6 FGF23 (ranging from −0.6 K to −1). Each *Fgf23* promoter construct was cotransfected with the *BBX* expression vector into MC3T3-E1 cells. FGF23 transcriptional activity was unchanged, regardless of the presence or absence of BBX (Supplementary Fig. 6a). Next, we analyzed the effect of BBX on enhancing FGF23 promoter activity using 1,25(OH)_2_D_3,_ which is a known FGF23 stimulator. *BBX* overexpression significantly suppressed 1,25(OH)_2_D_3_-induced FGF23 transactivation (Fig. 5a). The regulation of FGF23 transactivation by BBX was confirmed through *Bbx* knockdown. Transfection of the *Bbx* shRNA or treatment with 1,25(OH)_2_D_3_ partially induced FGF23 production in MC3T3-E1 cells; however, when the *Bbx* shRNA and 1,25(OH)_2_D_3_ were combined, FGF23 production was additively increased (Fig. 5b). BMSCs derived from *Bbx*^*−/−*^ mice also presented increased *Fgf23* expression compared with those derived from *Bbx*^*+/+*^ mice; however, a more significant increase its expression in *Bbx*^*−/−*^ mice was induced by 1,25(OH)_2_D_3_ (Fig. 5c). Furthermore, 1,25(OH)_2_D_3_ treatment markedly downregulated BBX protein levels (Fig. 5d). These results strongly suggest a negative regulatory effect of BBX on FGF23 expression and that 1,25(OH)_2_D_3_ enhances FGF23 transactivation by downregulating BBX.

### Physical interaction of BBX with VDR but not RXR

Flag-tagged *BBX* and *VDR* cDNAs were transfected into cells that were then treated with 1,25(OH)_2_D_3_, and immunoprecipitation was conducted to determine the functional significance of the relationship between BBX and VDR. As shown in Fig. 5e, BBX pulldown with a Flag antibody followed by VDR immunoblotting revealed that BBX interacted with VDR and that the interaction was further increased by VDR overexpression followed by 1,25(OH)_2_D_3_ treatment. An inverse pulldown experiment with a VDR antibody followed by BBX immunoblotting revealed that the BBX and VDR interaction was also increased by *VDR* overexpression and 1,25(OH)_2_D_3_ treatment. These results suggest that 1,25(OH)_2_D_3_ stimulation of VDR enhances its interaction with BBX. Notably, although the BBX and VDR interaction was increased by 1,25(OH)_2_D_3_, BBX protein levels were decreased by 1,25(OH)_2_D_3_ (Fig. 5e, input). The physical interaction of BBX with VDR was confirmed with endogenous proteins. The interaction between BBX and VDR was enhanced by 1,25(OH)_2_D_3_; however, BBX did not interact with RXR, regardless of the absence or presence of 1,25(OH)_2_D_3_ (Fig. 5f). These results suggest that the enhanced interaction of BBX with VDR, which is induced by 1,25(OH)_2_D_3_, may regulate the initial binding of the VDR-RXR heterodimer to the 1,25(OH)_2_D_3_ response element.

### Downregulation of BBX via proteasomal degradation

Our findings suggest that 1,25(OH)_2_D_3_ downregulates BBX protein levels. This decrease appears to occur posttranscriptionally, as 1,25(OH)_2_D_3_ treatment resulted in a notable increase in *Vdr* mRNA levels, whereas *Bbx* mRNA levels were not significantly altered (Fig. 5g). These findings indicate that 1,25(OH)_2_D_3_ downregulates the total BBX protein level without influencing *Bbx* gene transcription through translational repression or protein stability. Indeed, when protein synthesis was inhibited with cycloheximide (CHX), the decrease in BBX protein levels accelerated, with a half-life of 18 h after 1,25(OH)_2_D_3_ treatment, whereas endogenous BBX remained highly stable (Fig. 5h). Thus, 1,25(OH)_2_D_3_ treatment downregulates BBX protein expression by promoting its destabilization. Next, we determined whether BBX downregulation is caused by proteasome-mediated degradation. The cells were treated with MG-132, an inhibitor of proteasomal degradation, and the BBX protein level was measured. As shown in Fig. 5i, 1,25(OH)_2_D_3_ treatment partially downregulated BBX protein levels in the absence of *VDR* overexpression; however, *VDR* overexpression significantly downregulated BBX expression to a greater extent. Moreover, MG-132 treatment markedly increased the accumulation of the BBX protein, indicating that BBX undergoes proteasomal degradation in response to 1,25(OH)_2_D_3_. As protein ubiquitination is the primary mechanism for proteasomal degradation, BBX ubiquitination was assessed in response to 1,25(OH)_2_D_3_. Overexpressed Flag-tagged BBX was pulled down with a Flag antibody and probed with a ubiquitin antibody. As shown in Fig. 5j, the intensity of the ubiquitin signal was partially decreased by 1,25(OH)_2_D_3_ treatment, probably resulting from proteasomal degradation, but was noticeably increased by MG-132 treatment. Moreover, blocking BBX degradation with MG-132 treatment markedly inhibited FGF23 transactivation (Fig. 5k). These results suggest that 1,25(OH)_2_D_3_ induces the BBX–VDR interaction associated with the proteasomal degradation of BBX through ubiquitination, which results in FGF23 upregulation.

## Discussion

Hypophosphatemia occurs because of the disruption of phosphate homeostasis resulting from phosphate excretion and wasting in the kidney, malabsorption in the intestine, and malnutrition^2,43^. Hypophosphatemic bones contain an increased proportion of osteoid and decreased mature bone tissue, primarily resulting from under-mineralization. Several factors, including FGF23, have been well-established as key regulators of phosphate metabolism^3–5^. In the present study, we showed for the first time that *Bbx* deficiency causes hypophosphatemia through the upregulation of FGF23.

*Bbx*-deficient mice presented a reduced length, size, BMD, and BV of long bones four weeks after birth. In other bone regions, such as the frontal bone, spine, and iliac crest, in *Bbx*-deficient mice, the BV was low, but the BMD was normal or tended to be low. These morphological features appear to be associated with a small body size and low body weight. The phenotypic alterations of the long bones in both conventional and osteoblast/osteocyte-specific Bbx-deficient mice persisted for 8 weeks; however, the changes in some bone parameters did not reach statistical significance, indicating that the abnormal phenotype and parameters of the skeleton were partially ameliorated as growth progressed. This amelioration may occur because the demand for phosphate is reduced after the growth plate is closed, and bone turnover decreases in mature mice, such as 8-week-old mice^20,44^. Interestingly, the skeletal morphology and mineralization of long bones appeared normal at birth (P0). These results suggest that in *Bbx*-deficient mice, skeletal abnormalities initiate postnatally, become prominent during adolescence (at 4 weeks of age), and persist into adulthood (8 weeks of age). An analysis of the mechanical characteristics of bone in *Bbx*-deficient mice revealed that the MMI of the trabecular bone was significantly decreased in *Bbx*-deficient mice, indicating that the long bones of *Bbx*-deficient mice are less resistant to torsion. The fracture test indicated that the long bones of *Bbx*-deficient mice were mechanically weak and fragile. Therefore, the long bones of *Bbx*-deficient mice are characterized by small/short size, low mineral density, and weak mechanical strength.

The phenotypic abnormalities of bones in *Bbx*-deficient mice suggest an association with osteomalacia/rickets; however, these abnormalities are not as severe as those observed in individuals with calcipenic and hypophosphatemic rickets^20^. The phenotypic severity of bones in individuals with rickets may vary depending on the genes that are mutated^19,20^. The severity of the phenotypic alterations may be attributed to the functional and structural roles of the genes that are mutated. For example, DMP1 is a bone matrix protein, and its mutation may disrupt the normal arrangement of matrix proteins and calcium phosphate crystals^45^. Mutant *PHEX* does not degrade or cleave matrix extracellular phosphoglycoproteins or FGF23, which results in X-linked hypophosphatemic rickets^46,47^. Ectonucleotide pyrophosphatase/phosphodiesterase 1 (*ENPP*) mutation is closely associated with the phosphate concentration via the generation of pyrophosphate^48^; however, BBX functions as a transcriptional regulator, not a direct component of the bone matrix. Thus, it may affect only the expression of genes that control the bone composition. Unexpectedly, the expression of marker genes for osteoblast differentiation, including runt-related transcription factor 2 (*Runx2*), alkaline phosphatase (*Alp*), and bone sialoprotein (*Bsp*), was not decreased in the bones of *Bbx*-deficient mice. In contrast, the expression of genes related to osteocyte function, such as *Dmp1*, *Phex*, and *Sost*, was significantly decreased in *Bbx*-deficient mice. These results suggest that osteoblast differentiation is not substantially affected by *Bbx* deficiency; however, the late stage of osteoblast differentiation, such as the osteoblast–osteocyte transition or osteocyte function, may be affected by BBX. Similarly, osteoblast/osteocyte-specific *Bbx*-deficient mice also presented a reduced number of osteocytes. Nevertheless, the BFR and MAR were significantly decreased by *Bbx* deficiency. Although this discrepancy cannot be resolved, a reasonable expectation is that osteoblast function and differentiation processes from mesenchymal stem cells can proceed normally, but reduced osteoblast numbers may be attributed to a reduced BFR and MAR in *Bbx*-deficient mice. Moreover, the decrease in the number of osteoblasts appears to be associated with a decrease in the number of osteocytes. A compensatory mechanism may exist for *Bbx* mutation. BBX is an HMG box-containing protein that belongs to the TCF/LEF protein family of the Wnt signaling pathway^21^. Therefore, even if BBX is knocked out, other closely related proteins may at least partially compensate for the function of disrupted BBX.

Hypophosphatemia caused by *Bbx* disruption is caused by a decrease in the reabsorption of phosphate resulting from NPT2a and NPT2c downregulation in the renal proximal tubule, as well as decreased absorption due to the downregulation of NPT2b in the intestine. Moreover, the serum 1,25(OH)_2_D_3_ and PTH levels in *Bbx*-deficient mice were significantly lower than those in wild-type mice. Previous reports have indicated that excessive FGF23 downregulates NPT2a, NPT2b, and NPT2c, as well as PTH and 1,25(OH)_2_D_3_ production^9–15^. The expression and production of FGF23 were dramatically increased in both conventional and osteoblast/osteocyte-specific *Bbx*-deficient mice. These results suggest that BBX regulates the expression of FGF23, which in turn regulates the expression of sodium phosphate cotransporters and the production of PTH and 1,25(OH)_2_D_3_.

To date, the detailed regulatory mechanism of FGF23 expression is unknown^49,50^, although 1,25(OH)_2_D_3_, PTH, and other hormones induce or suppress FGF23 expression^50^. Moreover, mutations in *DMP1* and *ENPP* induce *FGF23* expression^51–53^; however, how they affect *FGF23* expression remains unclear. In addition, transcriptional repression has not been completely demonstrated. In the present study, *BBX* overexpression strongly inhibited FGF23 promoter transactivation, whereas *Bbx* knockdown enhanced FGF23 transactivation. These results suggest that BBX is a negative regulator of FGF23 transactivation. Recently, a pharmacological study indicated that peroxisome proliferator-activated receptor α (PPARα), a transcription factor, also negatively regulates *FGF23* expression in vitro^54^. However, the detailed regulatory mechanism in vitro and in vivo confirmation remains incomplete. Elucidating the relationship between BBX and PPARα in the negative transcriptional regulation of FGF23 would be interesting. Further studies on the negative regulation of FGF23 transactivation by BBX have shown that BBX interacts with VDR and undergoes proteasomal degradation through ubiquitination. In response to 1,25(OH)_2_D_3_, FGF23 transactivation was increased, and the interaction of BBX with VDR was increased; however, BBX protein levels were decreased by 1,25(OH)_2_D_3_ treatment or VDR overexpression. These results are interesting because increased protein–protein interactions are not typically accompanied by decreased protein levels; however, the 1,25(OH)_2_D_3_-mediated interaction of c-Myc with VDR and subsequent degradation of c-Myc have been observed in cancer cells^55^. Similarly, 1,25(OH)_2_D_3_-mediated BBX interactions with VDR may be associated with increased degradation of BBX. Although total BBX protein levels were decreased, VDR/BBX complex formation was increased by 1,25(OH)_2_D_3_. We hypothesize that 1,25(OH)_2_D_3_ stimulates VDR/BBX complex formation and that BBX may be degraded. Further studies focused on BBX degradation in response to 1,25(OH)_2_D_3_ are needed to elucidate BBX regulation; however, BBX degradation was detected using a proteasome inhibitor. BBX protein levels were increased by the addition of MG-132, a proteasome inhibitor, indicating that BBX undergoes proteasomal degradation in response to 1,25(OH)_2_D_3_. In addition, ubiquitination appears to be responsible for Bbx degradation. Although we showed that BBX interacts with VDR and undergoes ubiquitination-dependent degradation in response to 1,25(OH)_2_D_3_, additional experiments are needed to understand the BBX degradation mechanism in more detail.

**Fig. 6:**
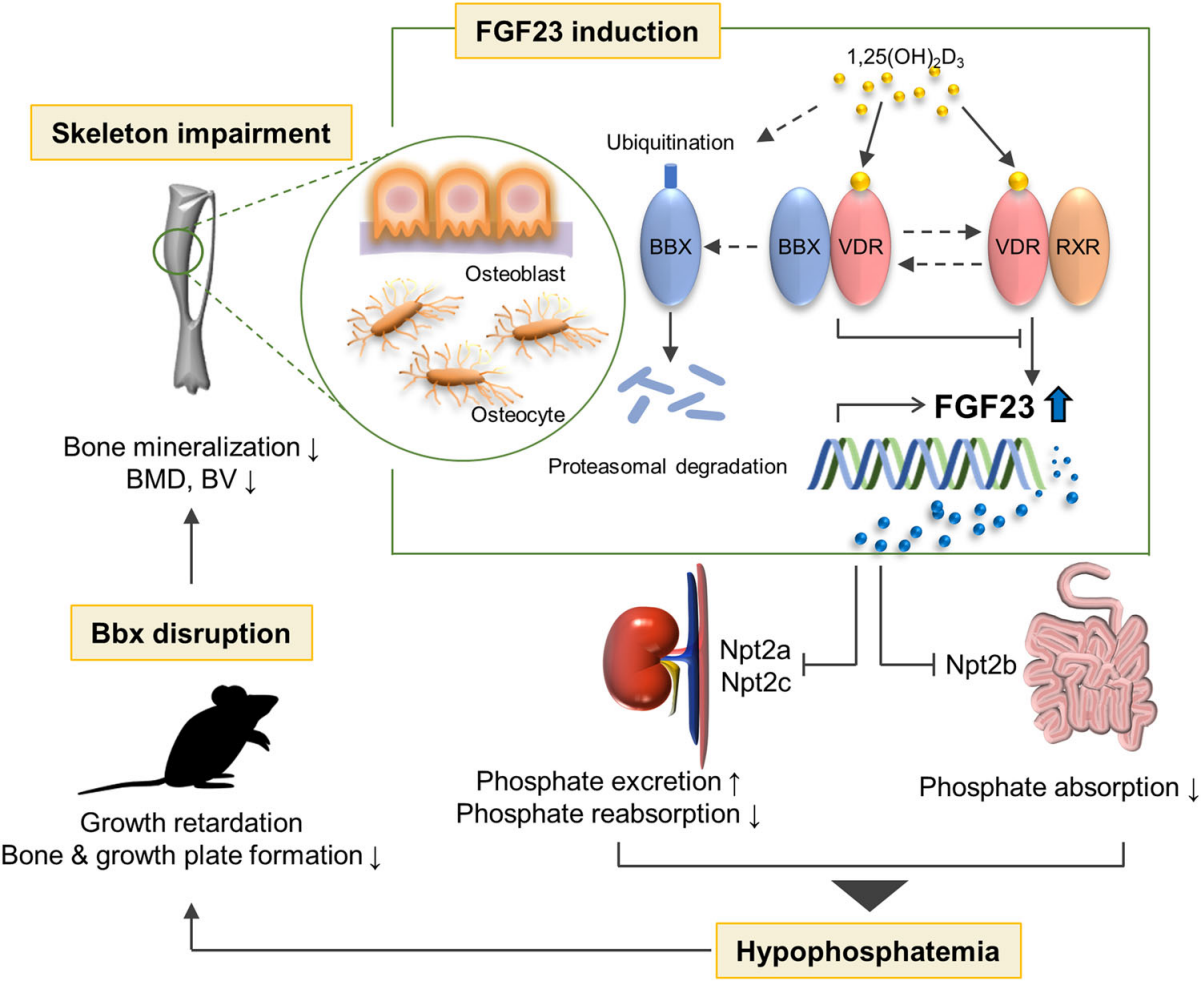
Schematic drawing illustrating the role of BBX in the transcription of FGF23 and its impact on phosphate homeostasis and bone development. BBX-deficient mice exhibit a phenotype characterized by delayed growth and skeletal abnormalities, including reduced bone mineralization, BMD, and BV. These abnormalities are linked to elevated FGF23 expression in osteoblasts and osteocytes, which leads to hypophosphatemia. The role of BBX in 1,25(OH)_2_D_3_-induced FGF23 expression is demonstrated, as ubiquitination and subsequent proteasomal degradation of BBX are required for this process. Therefore, BBX acts as a negative regulator of FGF23 expression and phosphate metabolism, ultimately influencing bone development and growth. FGF23 Fibroblast growth factor 23; BMD Bone mineral density; BV Bone volume.

In summary, we demonstrated that *Bbx* deficiency in the whole body, as well as specifically in osteoblasts/osteocytes, increases FGF23 expression, resulting in hypophosphatemia and subsequent bone abnormalities, including mechanical weakness, shortness, and a low BMD and BV. Furthermore, we found that the ubiquitination and subsequent proteasomal degradation of BBX are required for the 1,25(OH)_2_D_3_-mediated increase in FGF23 expression in vitro (Fig. 6). Therefore, BBX plays a negative role in FGF23 expression and phosphate metabolism, thereby affecting bone development.

## Supplementary information

Supplementary Information
