# Supplementary material for: The transcription factor BBX regulates phosphate homeostasis through the modulation of FGF23
Source: Exp Mol Med. 2024 Nov 1;56(11):2436–48. doi: 10.1038/s12276-024-01341-9 (PMC11612488; doi:10.1038/s12276-024-01341-9)
Supplement: Supplementary file 1 — Supplementary Information [file 12276_2024_1341_MOESM1_ESM.docx]

**Supplementary information**

**
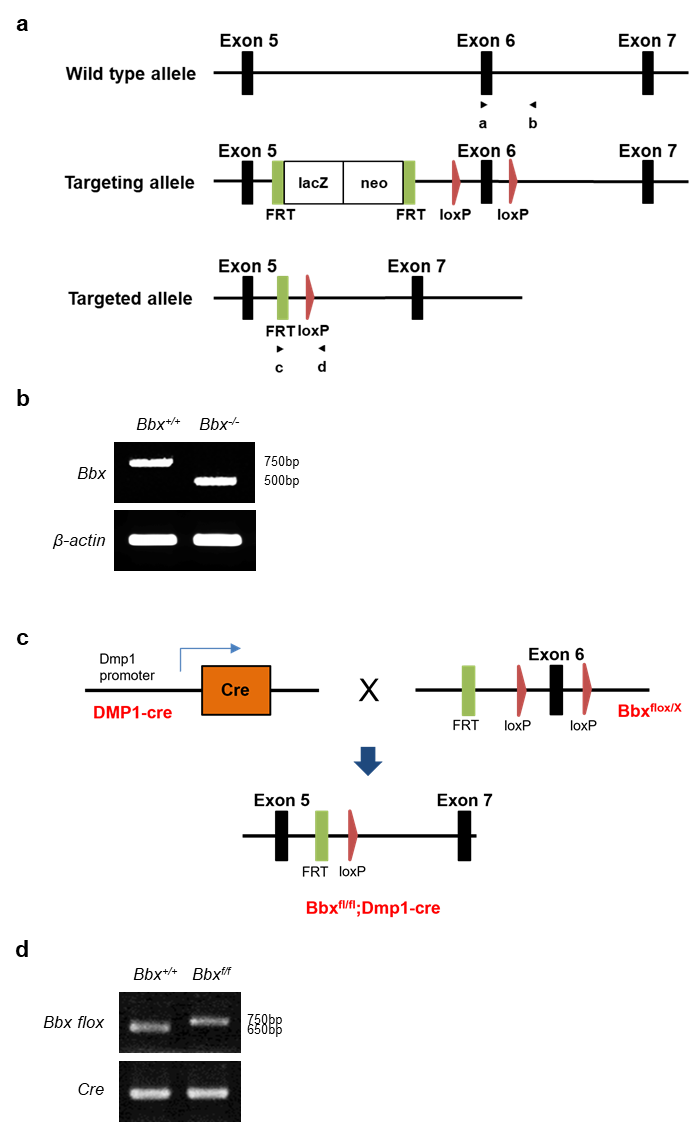
**

**Supplementary Fig. 1 Generation and confirmation of bobby sox homolog deficient (*Bbx^−/−^*) and *Bbx* conditional knockout mice.** **a** Targeting of the *Bbx* locus. The *Bbx* gene was knocked out using a targeting construct with lox P sites flanking exon 6. **b** PCR analysis of *Bbx* gene expression in four-week-old *Bbx^+/+^* and *Bbx^−/−^* mice. **c** Diagram of *Cre*-mediated *Bbx* deletion. **d** Representative PCR genotyping result for *Bbx* conditional knockout mice. β-actin was used as a loading control.


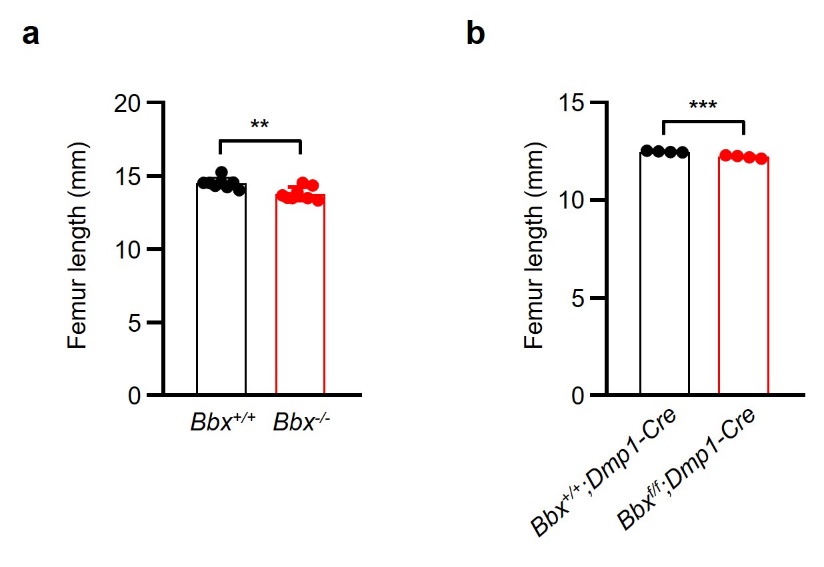


**Supplementary Fig 2. Micro-computed tomography analysis of the femoral bone in 8-week-old mice. a** Femur lengths of 8-week-old *Bbx^+/+^* and *Bbx^−/−^* mice were measured (n = 8). **b** Femur lengths of 8-week-old *Bbx^+/+^;Dmp1-Cre* and *Bbx^f/f^;Dmp1-Cre* mice were measured (n = 4). Data are presented as means ± SD. ***p* < 0.01, ****p* < 0.001 as determined by unpaired Student’s *t*-test between the two indicated genotypes. Male mice were used for the experiments.

**
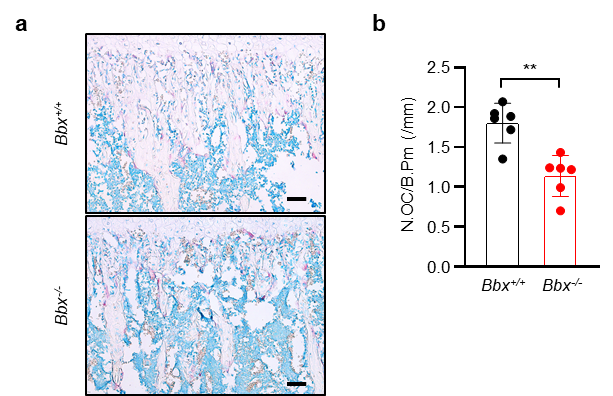
**

**Supplementary Fig. 3 Tartrate-resistant acid phosphatase (TRAP) staining and osteoclast counting.** **a** Tibial sections of 4-week-old *Bbx^+/+^* and *Bbx^−/−^* mice were stained using TRAP. TRAP-positive cells (red) indicate osteoclasts. Scale bars, 50 μm. **b** The number of osteoclasts per bone perimeter (N.OC/B.Pm) was counted (n = 6). Data are presented as means ± SD. ***p* < 0.01 as determined by an unpaired Student’s *t*-test between the two indicated genotypes.


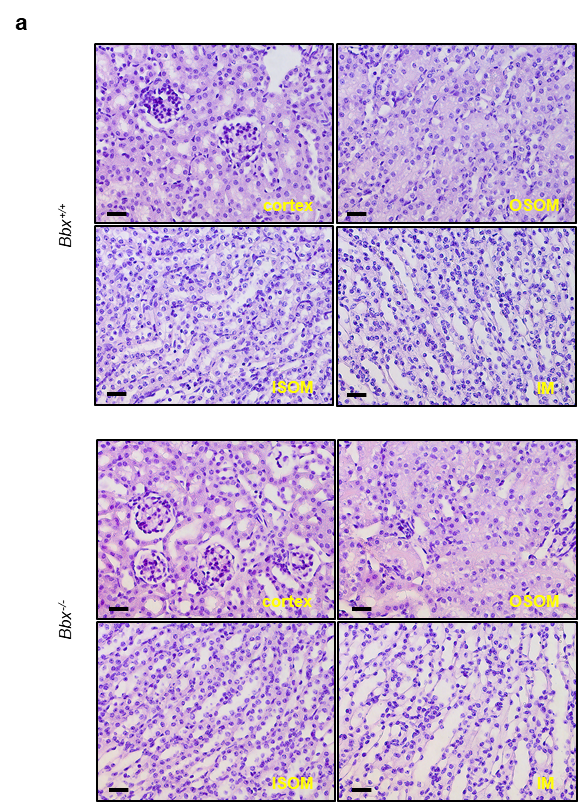


**Supplementary Fig. 4 Representative images of H&E staining of kidneys from 4-week-old *Bbx^+/+^* and *Bbx^−/−^* mice.** Scale bars, 25 μm. OSOM, outer stripe of the outer medulla; ISOM, inner stripe of the outer medulla; IM, inner medulla. Representative images from three individuals are shown.

**
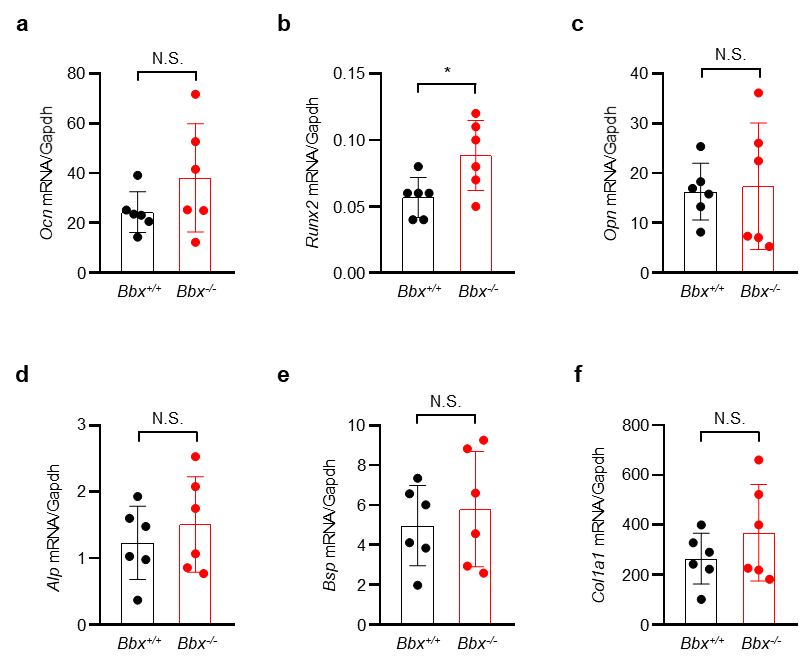
**

**Supplementary Fig. 5** **Quantitative RT-PCR reaction analyses of the mRNA levels of osteoblast marker genes; *Ocn* (a), *Runx2* (b), *Opn* (c), *Alp* (d), *Bsp* (e), and *Col1a1* (f) from the femurs of 4-week-old *Bbx^+/+^* and *Bbx^−/−^*** **mice (n = 6).** The values are normalized to *Gapdh*. Data are presented as means ± SD. **p* < 0.05 as determined by an unpaired Student’s *t*-test between the two indicated genotypes. N.S. indicates no significant difference compared with wild-type mice.

**
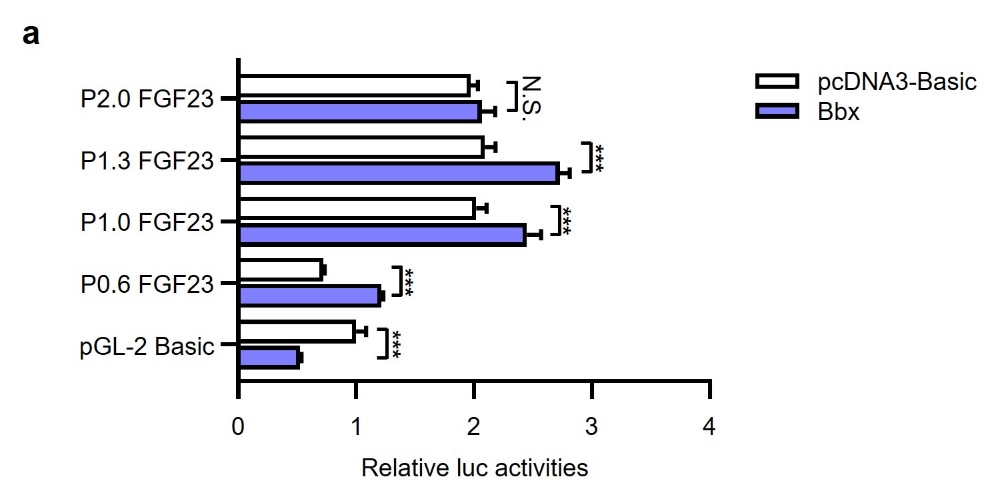
**

**Supplementary Fig. 6** **Effect of BBX on *Fgf23* promoter transactivation.** **a** MC3T3-E1 cells were co-transfected with *BBX* cDNA and *FGF23* promoter-firefly luciferase plasmids, incubated for 24h, and luciferase-firefly activity was measured. Data are presented as means ± SD. ****p* < 0.001 as determined by two-way ANOVA. N.S. indicates no significant difference compared with each of the two groups.

**Supplementary Tables**

**Supplementary Table 1. Primer sequences.**

| Name | Sequence to 5’ to 3’ | | Species |
| --- | --- | --- | --- |
| Fgf23 | F | TTTCCCAGGTTCGTCTAGG | Mouse |
|  | R | CTCGCAGGTGACTCTCAG |  |
| Npt2a | F | GGCTCCAACATTGGCACTACCA | Mouse |
|  | R | ACCACAGTAGGATGCCCGAGAT |  |
| Npt2b | F | GGCAACACCTTGAGGAGTTCTC | Mouse |
|  | R | GAACCAGCGATACTTGGCAGAG |  |
| Npt2c | F | GCGGTATTACCAGCAACACCAC | Mouse |
|  | R | TGTCCTCCTCTGGAGATGCTGA |  |
| Phex | F | CTGGCTGTAAGGGAAGACTTCC | Mouse |
|  | R | GCTCCTAAAAGCACAGCAGTGTC |  |
| Dmp1 | F | GAAAGCTCTGAAGAGAGGACGG | Mouse |
|  | R | CCTCTCCAGATTCACTGCTGTC |  |
| Sost | F | TCCTCCTGAGAACAACCAGACC | Mouse |
|  | R | TCTGTCAGGAAGCGGGTGTAGT |  |
| Ocn | F | GCAATAAGGTAGTGAACAGACTCC | Mouse |
|  | R | CCATAGATGCGTTTGTAGGCGG |  |
| Runx2 | F | CCTGAACTCTGCACCAAGTCCT | Mouse |
|  | R | TCATCTGGCTCAGATAGGAGGG |  |
| Opn | F | GCTTGGCTTATGGACTGAGGTC | Mouse |
|  | R | CCTTAGACTCACCGCTCTTCATG |  |
| Alp | F | CCAGAAAGACACCTTGACTGTGG | Mouse |
|  | R | TCTTGTCCGTGTCGCTCACCAT |  |
| Bsp | F | AATGGAGACGGCGATAGTTCCG | Mouse |
|  | R | GGAAAGTGTGGAGTTCTCTGCC |  |
| Col1a1 | F | CCTCAGGGTATTGCTGGACAAC | Mouse |
|  | R | CAGAAGGACCTTGTTTGCCAGG |  |
| Bbx | F | AAACGACCAAAACGGAAGTG | Mouse |
|  | R | GCTGCTCAGGTGACTCATCA |  |
| Vdr | F | GCTCAAACGCTGCGTGGACATT | Mouse |
|  | R | GGATGGCGATAATGTGCTGTTGC |  |

**Supplementary Table 2.** **Micro-computed tomographic analysis of the frontal bone, spine, and illium.**

| **Bone** | **Genotype** | **BMD (g/mm^3^)** | **BV (mm^3^)** | **BV/TV (%)** | **Tb.Th (mm)** | **Tb.N (1/mm)** | **Tb.Sp (mm)** |
| --- | --- | --- | --- | --- | --- | --- | --- |
| Frontal bone | *Bbx^+/+^* | 0.185(0.017) | 0.239(0.019) | 23.147(1.888) | 0.063(0.005) | 3.669(0.295) | 0.115(0.006) |
|  | *Bbx^−/−^* | 0.175(0.031) | 0.225(0.029) | 21.769(2.852) | 0.062(0.005) | 3.520(0.198) | 0.115(0.003) |
| Spine | *Bbx^+/+^* | 0.021(0.0003) | 0.064(0.010) | 8.639(0.993) | 0.041(0.003) | 2.107(0.197) | 0.195(0.012) |
|  | *Bbx^−/−^* | 0.021(0.0002) | 0.053(0.004)* | 7.952(0.718) | 0.038(0.0004) | 2.070(0.170) | 0.194(0.007) |
| Ilium | *Bbx^+/+^* | 0.065(0.008) | 0.026(0.006) | 3.974(0.562) | 0.045(0.001) | 0.087(0.108) | 0.320(0.019) |
|  | *Bbx^−/−^* | 0.059(0.007) | 0.016(0.003)** | 3.386(0.665) | 0.043(0.002) | 0.786(0.178) | 0.323(0.015) |

An unpaired Student’s *t*-test was performed. **p* < 0.05, ***p* < 0.01.

**Supplementary Table 3. Bone parameters of the femur in 8-week-old *Bbx^−/−^*and *Bbx^f/f^;Dmp1-cre* mice.**

| **Bone** | **Genotype** | **BMD (g/mm^3^)** | **BV (mm^3^)** | **BV/TV (%)** | **Tb.Th (mm)** | **Tb.N (1/mm)** | **Tb.Sp (mm)** | **MMI (mm^4^)** |
| --- | --- | --- | --- | --- | --- | --- | --- | --- |
| Trabecular bone | *Bbx^+/+^* | 0.174(0.015) | 0.447(0.065) | 12.303(1.673) | 0.046(0.003) | 2.653(0.318) | 0.201(0.009) | 0.282(0.014) |
|  | *Bbx^−/−^* | 0.174(0.030) | 0.375(0.090) | 11.570(2.853) | 0.044(0.006) | 2.606(0.469) | 0.207(0.017) | 0.198(0.016)** |
|  | *Bbx^+/+^;Dmp1-cre* | 0.195(0.004) | 1.382(0.403) | 13.780(3.152) | 0.069(0.012) | 1.999(0.203) | 0.438(0.037) | 1.128(0.289) |
|  | *Bbx^f/f^;Dmp1-cre* | 0.190(0.003) | 0.786(0.159)* | 9.187(1.619)* | 0.059(0.002) | 1.549(0.240)* | 0.403(0.041) | 0.598(0.140)* |

| **Bone** | **Genotype** | **BMD (g/mm^3^)** | **BV/TV (%)** | **Ct.Th (mm)** | **Ct.Ar (mm^2^)** | **Ecc** | **Ct.Cs.Th (mm)** | **MMI (mm^4^)** |
| --- | --- | --- | --- | --- | --- | --- | --- | --- |
| Cortical bone | *Bbx^+/+^* | 0.605(0.016) | 74.386(0.786) | 0.155(0.005) | 0.686(0.038) | 0.715(0.016) | 0.129(0.007) | 0.382(0.043) |
|  | *Bbx^−/−^* | 0.883(0.021) | 72.826(0.957)* | 0.144(0.006)* | 0.615(0.042)* | 0.385(0.180)* | 0.121(0.006) | 0.317(0.039)* |
|  | *Bbx^+/+^;Dmp1-cre* | 0.966(0.014) | 35.421(2.226) | 0.135(0.012) | 0.688(0.074) | 0.735(0.011) | 0.135(0.012) | 0.369(0.056) |
|  | *Bbx^f/f^;Dmp1-cre* | 0.988(0.016) | 33.676(0.374) | 0.108(0.014)* | 0.632(0.015) | 0.718(0.017) | 0.128(0.002) | 0.329(0.012) |

An unpaired Student’s *t*-test was performed. **p* < 0.05, ***p* < 0.01.

Numbers of each groups; *Bbx^+/+^* (n = 5), *Bbx^−/−^* (n = 5), *Bbx^+/+^;Dmp1-Cre* (n = 4), *Bbx^f/f^;Dmp1-Cre* (n = 4).
